# Application and Analysis of the Folin Ciocalteu Method for the Determination of the Total Phenolic Content from *Limonium Brasiliense* L.

**DOI:** 10.3390/molecules18066852

**Published:** 2013-06-10

**Authors:** Andressa Blainski, Gisely Cristiny Lopes, João Carlos Palazzo de Mello

**Affiliations:** Programa de Pós Graduação em Ciências Farmacêuticas, Universidade Estadual de Maringá, Av. Colombo, 5790, BR-87020-900, Paraná, Brazil; E-Mails: andressa_blainski@yahoo.com.br (A.B.); giselycl@yahoo.com.br (G.C.L.)

**Keywords:** *Limonium brasiliense*, methodology optimization, total polyphenols, UV/Vis spectrophotometric validation, Folin-Ciocalteu

## Abstract

*Limonium brasiliense* is a common plant on the southern coast of Brazil. The roots are traditionally used for treatment of premenstrual syndrome, menstrual disturbances and genito-urinary infections. Pharmaceutical preparations obtained from its roots and used for these purposes were marketed in Brazil in the 1980s and 1990s. Currently, the Brazilian Drug Agency (National Health Surveillance Agency, ANVISA) has canceled the registration of these products, and their use was discontinued because of a lack of studies to characterize the plant raw material and ensure the effectiveness and safety of its use. The aim of the present study was to develop and validate an analytical method to determine the content of total polyphenols (TP) in an extract from *L. brasiliense* roots, by the UV/Vis spectrophotometric method. *L. brasiliense* roots were extracted in acetone:water (7:3, v/v-10% w/v). The crude extract was used to develop a method for TP assay. The method was validated according to national and international guidelines. The optimum conditions for analysis time, wavelength, and standard substance were 30 min, 760 nm, and pyrogallol, respectively. Under these conditions, validation by UV/Vis spectrophotometry proved the method to be linear, specific, precise, accurate, reproducible, robust, and easy to perform. This methodology complies with the requirements for analytical application and to ensure the reliability of the results.

## 1. Introduction

Phenolics include simple phenols, phenolic acids (benzoic and cinnamic acid derivatives), coumarins, flavonoids, stilbenes, hydrolyzable and condensed tannins, lignans, and lignins. These compounds are among the most widely occurring secondary metabolites in the plant kingdom, acting mainly as phytoalexins, attractants for pollinators, contributors to plant pigmentation, antioxidants, and protective agents against UV light, among others [1]. Among the many varieties of natural phenolic compounds, the procyanidins are an important subgroup. These substances are composed of oligomers and polymers that consist of catechin and/or epicatechin units linked mainly through C4→C8 and/or C4→C6 bonds (B-type). The flavan-3-ol units can be doubly linked by a C4→C8 bond and/or an additional ether bond from O7→C2 (A-type) [2,3].

Although quantitative determination of polyphenols is hampered by their structural complexity and diversity, several methods have used to determine polyphenols in plant extracts [4,5,6,7]. Assuming that quantification of individual polyphenols does not adequately reveal the proportion of polymeric procyanidins, then spectrophotometry in the ultraviolet region may be a useful tool to help resolve this problem [8,9].

Colorimetric reactions are widely used in the UV/VIS spectrophotometric method, which is easy to perform, rapid and applicable in routine laboratory use, and low-cost [10]. However, it is important that colorimetric assay need to use a reference substance, then this method mensures the total concentration of phenolic hydroxyl groups in the plant extract. Polyphenols in plant extracts react with specific redox reagents (Folin-Ciocalteu reagent) to form a blue complex that can be quantified by visible-light spectrophotometry [11]. The Folin-Ciocalteu method is described in several pharmacopoeias [12,13]. The reaction forms a blue chromophore constituted by a phosphotungstic-phosphomolybdenum complex [11,14], where the maximum absorption of the chromophores depends on the alkaline solution and the concentration of phenolic compounds [11]. However, this reagent rapidly decomposes in alkaline solutions, which makes it necessary to use an enormous excess of the reagent to obtain a complete reaction. This excess can result in precipitates and high turbidity, making spectrophotometric analysis impossible. To solve this problem, Folin and Ciocalteu included lithium salts in the reagent, which prevented the turbidity [15]. The reaction generally provides accurate and specific data for several groups of phenolic compounds, because many compounds change color differently due to differences in unit mass [16] and reaction kinetics [15].

Many studies have discussed the use of the Folin-Ciocalteau reagent to determine polyphenols, and the general or specific value of the method, because some specific details may be modified [9,14,17,18]. *Limonium brasiliense* (Boiss.) Kuntze (Plumbaginaceae) is a perennial herb that occurs in Argentina, Uruguay, and on the southern coast of Brazil. It is popularly known in Brazil as “baicuru” or “guaicuru”, and is traditionally used to treat premenstrual syndrome, menstrual disorders and genitourinary infections [19,20,21]. Pharmaceutical preparations obtained from its roots and used for these purposes were marketed in Brazil in the 1980s and 90s [22,23]. Currently, the Brazilian Drug Agency (National Health Surveillance Agency, ANVISA) has canceled the registration of these products, and their use was discontinued because of a lack of studies to characterize the plant raw material and ensure the effectiveness and safety of its use.

The few studies of *L. brasiliense* describe its biological activities, including bacteriostatic, anti-inflammatory and antioxidant, which were related to the presence of condensed and hydrolyzable tannins, leucoanthocyanins, flavonoids, β-sitosterol, saponins and coumarin in the extracts evaluated [20,21,22,23,24].

This report describes the first of a series of chemical studies of *L.* *brasiliense*, aimed at characterizing the plant drug and standardization and normalization of the content of polyphenols in the extracts. This study deals with the optimization of a method for determination of total polyphenols and an evaluation of possible variations of the Folin-Ciocalteau method applied to the crude extract.

## 2. Results and Discussion

### 2.1. Method Optimization

**Figure 1:**
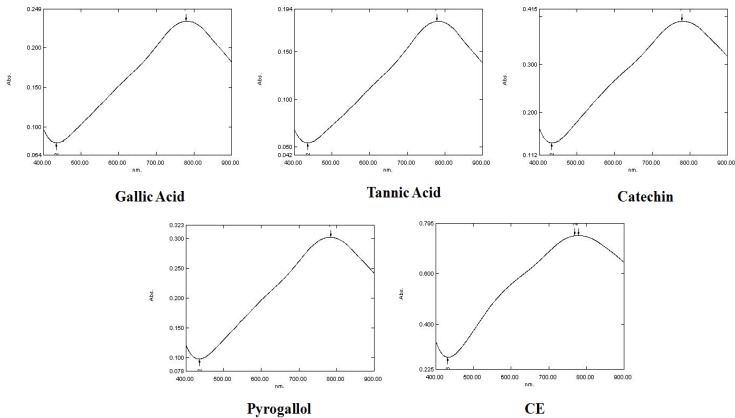
Absorption spectra (400 to 900 nm) of reference compounds (gallic acid, tannic acid, catechin and pyrogallol) and CE from *L. brasiliense* by Folin-Ciocalteu reaction after 30 min.

According to Glasl [16], each plant has a characteristic chemical composition, with uniform phenolic groups present in the same species. Similar chemical structures may show the same chemical interactions with specific reagents during the reaction [16]. All the reference substances used show approximately the same spectra as the CE from *L. brasiliense*, so it is not possible to determine the best reference compound for this extract by means of their spectra (Figure 1).

The percentage increase in absorbance of each solution was compared at the wavelengths of 691, 715, 760, and 800 nm. The statistical analysis indicated a significant increase in absorbance at 760 nm compared to data for 691 and 715 nm for all solutions (gallic acid, F_60,3_ = 1,081.9, *P* < 0.001; tannic acid, F_60,3_ = 1,213.8, *P* < 0.001; catechin, F_60,3_ = 1,033.2, *P* < 0.001; pyrogallol, F_60,3_ = 1,337.4, *P* < 0.001; CE from *L. brasiliense*, F_60,3_ = 4,910.0, *P* < 0.001). The absorbance at 800 nm was higher than at the other wavelengths; however, this increase was not statistically significant for the ratio at 760 nm, which suggests that this parameter is stable, as determined by the European Pharmacopoeia.

Using the wavelength of 760 nm, which appeared to be best suited to produce maximum absorption of the substances under study, the next step was to determine the reaction kinetics with respect to the time period prior to the spectrophotometric measurement. The increase in percentage absorbance of each solution relative to the time periods of 10, 20, 30 and 40 min was determined. These data showed that the Folin–Ciocalteu reaction was stable during the period analyzed, since after 30 min the absorbance increased less than 5% of the value at 10 min, and did not decrease between 30 and 40 min. The statistical analysis indicated no significant diference in relation at the time (gallic acid, F_8,3_ = 0.0108, *P* = 0.9983; tannic acid, F_8,3_ = 0.0483, *P* = 0.9849; catechin, F_8,3_ = 0.0042, *P* = 0.9996; pyrogallol, F_8,3_ = 0.0630, *P* = 0.9777). These results suggested a period of 30 min for the spectrophotometric measurement of the CE.

**Figure 2:**
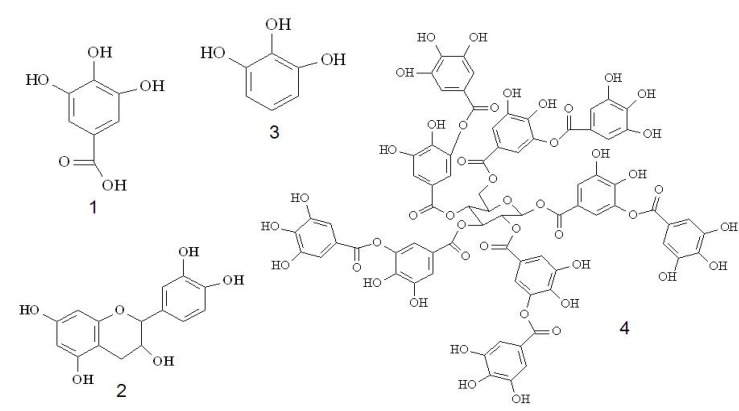
Chemical structures of reference compounds: (1) gallic acid (C_7_H_6_O_5_), (2) catechin (C_15_H_14_O_6_), (3) pyrogallol (C_6_H_6_O_3_) and (4) tannic acid (C_76_H_52_O_46_).

With respect to the standard, different specific absorptivities (760 nm; 30 min) were observed for each reference compound: A^1%^_gallic acid_ = 970.8 ± 31.46; A^1%^_tannic acid_ = 830.0 ± 39.29; A^1%^_catechin_ = 1,188.7 ± 62.50 and A^1%^_pyrogallol_ = 1,551.8 ± 93.95. These results showed that the intensity of the reaction of phenolic compounds with Folin–Ciocalteu reagent follows the classical principle of structure-activity relationship, in which the order of activity is proportional to the availability of hydroxyl groups present on the aromatic ring and influenced by groups that can decrease or increase the reductive potential of the molecule [25]. Of the substances in this study (Figure 2), pyrogallol has the largest number of hydroxyl groups proportional to its molar mass. In addition, pyrogallol has only one ring and no substituted groups, forming a three-dimensional structure with a hydroxyl that is less influenced by electronic interactions such as steric or resonance effects. Pyrogallol therefore has the highest specific absorptivity and is probably the best reference substance for the determination of TP. Also, pyrogallol indirectly represents the reduction potential of the plant drug, considering the total availability of hydroxyls linked to the aromatic ring. The method was optimized for an extract of *Caesalpinia peltophoroides* Benth., and similar results were obtained [18].

### 2.2. TP of CE from L. brasiliense and Calibration Curve of Pyrogallol Standard

The calibration equation was y = 0.14073x + 0.0044 (n = 5, r^2^ = 0.996) for pyrogallol. The relative standard deviations (RSDs) of the slopes were ≤5% for the analyte (n = 5). Table 1 shows the back-fit calculations for curve data for the standard used in the validation runs, as well as the precision and accuracy of the back-fit calculations.

The specific absorptivity is the absorbance of a solution in a concentration of 1 g/100 mL (1%-10,000 µg/mL). The specific absorptivity of pyrogallol was calculated using the linear equation (y = 0.14073x + 0.0044), obtaining a value of 1,407.3. Therefore, the mean absorbance for CE from *L. brasiliense* was (0.550 ± 0.03 [5.38]), and TP expressed as pyrogallol was 23.5%.

### 2.3. Linearity

**Figure 3:**
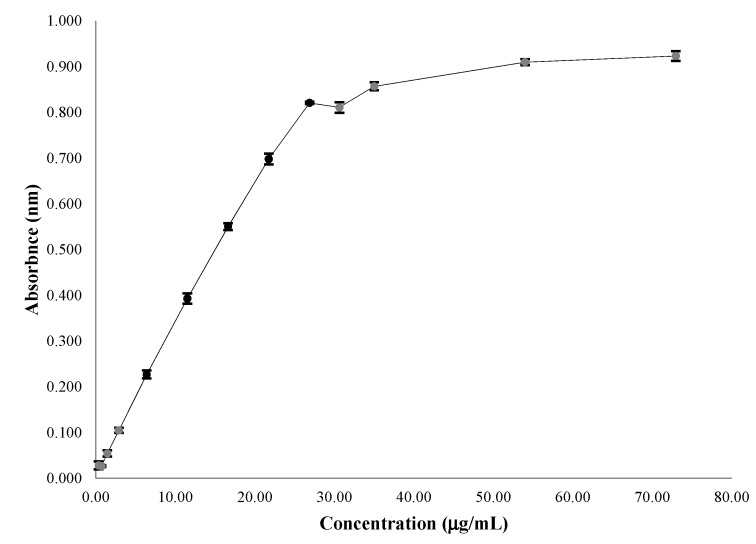
Absorbance *versus* concentration curve in the analysis of TP in CE from *L. brasiliense*; linearity (6.40 to 26.88 µg/mL).

Based on 1/x weighted linear regression analysis, the responses for the CE in related concentration ranges were linear (Figure 3). The data obtained in the linearity test for CE and pyrogallol appear in Table 1.

**Table 1:** Statistical data for the regression equations of the calibration curve for pyrogallol, and linearity test and specificity test for TP of CE from *L. brasiliense*.

Regression analysis	Calibration curve for pyrogallol	Linearity test for CE	Specificity test for CE
Slope (SE)	0.1407 (0.00225)	0.029 (0.00040)	0.031 (0.00030)
Intercept (SE)	0.0044 (0.0078)	0.046 (0.00010)	0.133 (0.0008)
Regression coefficient (R^2^)	0.996	0.996	0.997
Calculated F-value (critical F-value)	3.30 (3.71)	0.26 (3.71)	1.12 (3.71)
Sum of pure error	0.0990	0.0012	0.0009
Lack of fit error	0.0097	0.0010	0.0008
Analysis of variance	F_1,13_ = 3901.2, *P* < 0.001	F_1,13_ = 3999.8, *P* < 0.001	F_1,13_ = 5880.8, *P* < 0.001
CL slope	0.1359; 0.1456	0.0286; 0.0307	0.0303; 0.0320
CL intercept	−0.0125; 0.0213	0.0284; 0.0652	0.1173; 0.1491

SE = standard error; CL = confidence limit

### 2.4. Specificity

**Figure 4:**
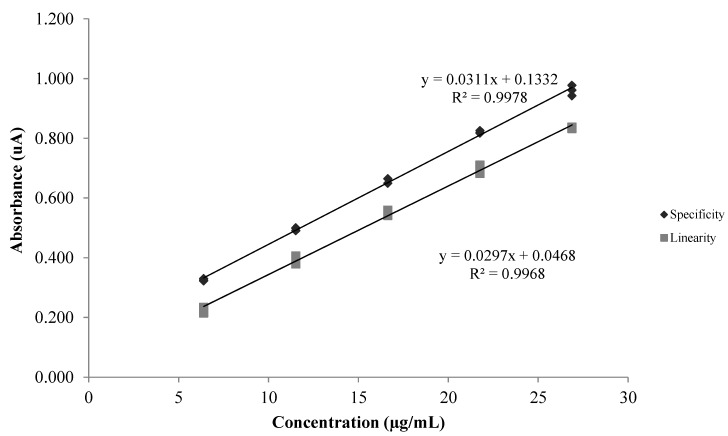
Linearity curve and specificity curve, with correlation coefficient (R^2^) and linear equation, for TP of CE from *L. brasiliense.*

Analysis of the results of the specificity test indicated that the conditions were satisfactory (Table 1). In the case of complex matrices, if the matrix without the analyte is not available, the effects of the matrix system can be tested by comparing the slopes of linearity and specificity [26,27,28]. If the curves are parallel, we can state that the method is specific [27]. The specificity of the method for CE was confirmed by superimposing the analytical curves (Figure 4), because the slopes of the linear equations (linearity and specificity, see Table 1) were very similar (about 6.5% difference).

### 2.5. Limits of Detection and Quantification

The quantification range was from 3.68 to 26.88 µg/mL (absorbance from 0.105 to 0.834), because linearity was maintained (R^2^ = 0.996). Based on the linear equation from the test for linearity, the LOQ was 3.34 µg/mL. According to the Lambert-Beer law, for a given concentration of the sample, the absorbance is not proportional to the concentration [29]. The LOD was 1.84 µg/mL (absorbance of 0.055), because there was a significant difference between this concentration and the next-lower concentration (0.92 µg/mL; t_1,4_ = −5.7, *P* < 0.001). Based on the linear equation from the linearity test, LOD was 1.0 µg/mL. The LOD and LOQ determined by the linear equation gave different results from those observed experimentally. These differences suggest be by linear regression from linearity curve providing a satisfactory result, which reduces the standard deviation of the *y* intercept, which is directly proportional to both limits. The experimental determination may be affected by other interfering factors, such as human manipulation and differences in the standard conditions among laboratories.

### 2.6. Precision

The repeatability and the intermediate precision for CE were 0.552 ± 0.027 [4.98%] and 0.530 ± 0.003 [0.65%], respectively, with no significant difference between them (t_1,7_ = 1.34, *P* = 0.22). Therefore, the proposed method is adequately precise.

### 2.7. Accuracy

The result for the accuracy test showed a percentage recovery of 101.6, 90.2 and 89.9%, respectively, for the lowest, intermediate and highest levels. These percentages were within the range of 85%–115% established in published reports. This indicates that the method has good accuracy for determining TP in CE from *L. brasiliense*.

### 2.8. Robustness

The change in Na_2_CO_3_ concentration in the robustness assay took into account data from the literature: 7.5% in the Brazilian Pharmacopeia [12], 14:05% in Glasl [16], and 10.75% in the European Pharmacopoeia [13]. The values for absorbance measured after changing the Na_2_CO_3_ concentration and the natural light incidence in the Folin–Ciocalteu reaction were 0.530 ± 0.01 [1.82%]; 0.530 ± 0.005 [0.89%], and 0.531 ± 0.01 [1.81%], respectively, without protection from light; Na_2_CO_3_ solution 7.5% (w/v); and Na_2_CO_3_ solution 14.06% (w/v); pH 11.0 for both solutions. Comparison of these results with those obtained in the linearity assay indicated no significant difference between means (F_3,8_ = 4.2, *P* = 0.05). However, there was a tendency for the proposed conditions and validated by method [protection from light and Na_2_CO_3_ solution 10.75% (w/v)].

## 3. Experimental

### 3.1. Standards and Chemicals

All chemicals were analytical-reagent grade and the water was distilled. The chemicals included acetone (Synth^®^, Diadema, Brazil), 2 *N* Folin-Ciocalteu reagent (Dinâmica^®^, Diadema, Brazil), anhydrous sodium carbonate (Synth^®^), pyrogallol (Fluka, St. Louis, MO, USA), gallic acid (Sigma-Aldrich^®^, St. Louis, MO, USA), tannic acid (Acros), and catechin (Sigma-Aldrich).

### 3.2. Plant Material

Roots of *L. brasiliense* were collected in Rio Grande, state of Rio Grande do Sul, Brazil (S 32° 09' 22''/W 52° 06' 01'') in May 2006. A voucher specimen was deposited at the Herbarium of the Federal University of Rio Grande under number HURG-004208. IBAMA-SISBIO gave permission for this collection under a license granted to João Carlos Palazzo de Mello (No. 11995-2, 05/07/2007).

### 3.3. Extraction

The plant material was washed with water to remove soil, dried in a circulating-air oven (37 ± 2 °C) and powdered in a hammer mill (Tigre ASN-5; mean diameter 0.42 mm). The milled roots (630 g) were extracted in 6.3 L of 7:3 acetone:water (v/v) by turbo-extraction (Ultra-Turrax^®^, Ika^®^ Works, Wilmington, North Carolina, USA, UTC 115KT, 30 min, <40°C). Next the extractive solution was filtered, washed with 7.2 L of 7:3 acetone:water (v/v), concentrated in a rotavapor under reduced pressure, lyophilized (Martin Christ Alpha 1-4) to yield a crude extract (CE, 272 g) and stored at −20 °C.

### 3.4. Method Optimization and Standardization for Determination of Total Polyphenols (TP)

For the optimization and standardization of the spectrophotometric method using the Folin-Ciocalteau reagent, three parameters were analyzed: (1) reaction kinetics, (2) maximum absorption wavelength, and (3) the standard that best characterizes the CE of *L. brasiliense*. The method utilized for the test was a colorimetric assay, based on general procedures recommended by the European Pharmacopoeia [9] for determination of total tannins, with slight modifications. We used a solution of 10.75% anhydrous sodium carbonate, corresponding to 29% sodium carbonate decahydrate.

For the selection of a reference chemical standard, stock solutions of each standard (gallic acid, tannic acid, catechin and pyrogallol, 20 µg/mL) and CE (300 µg/mL) were prepared in distilled water. Two milliliters of each solution was transferred to a 25 mL volumetric flask containing distilled water (10 mL) and Folin-Ciocalteu reagent (1 mL) [9]. The volume was completed with 10.75% anhydrous sodium carbonate (w/v), resulting in final concentrations of 1.6 µg/mL of each standard and 24 µg/mL of CE. The samples were scanned in a UV/Vis spectrophotometer (Shimadzu PC-1650, Kyoto, Japan) beginning 10 to 40 min after the addition of the sodium-carbonate solution, and scanning from 400 to 900 nm at intervals of 2 min between each reading, to determine the spectra. Distilled water was used as a blank, with a quartz cell (1 cm path length). 

The kinetic reaction was evaluated (ANOVA) by comparing the percentage increase in absorbance of each solution, for the wavelengths of 691 [16], 715 [13], 760 [12], and 800 nm. This percentage increase was calculated by dividing the difference in absorbance between two wavelengths by the mean absorbance of the shorter wavelength and multiplying by 100.

The reaction times were observed and compared statistically, using the percentage increase between the reaction times (10, 20, 30, and 40 min) at 760 nm. The specific absorptivity of each standard was calculated based on the Lambert-Beer Law [29].

### 3.5. Analytical Method Validation

For validation of the analytical method, the guidelines established by the ICH (International Conference on the Harmonization of Technical Requirements for the Registration of Pharmaceuticals for Human Use) and by Brazilian regulation RE No. 899/2003 of ANVISA were employed [28,30].

### 3.6. Method for TP Analysis of CE from L. brasiliense and Calibration Curve of Pyrogallol Standard

All extraction and dilution operations were protected from light. *CE solution*: the CE (26.0 mg) was transferred to a 25 mL volumetric flask and diluted with water, and 5 mL of this stock CE solution was diluted to 25 mL with water. Then, a mixture was prepared with 2 mL of this solution, 1 mL of 2 *N* Folin-Ciocalteu reagent, and 10 mL of water, and volumetrically diluted to 25 mL with 10.75% w/v anhydrous sodium carbonate (w/v)*.* After 30 min, the absorbance was measured at 760 nm, using water as the compensation liquid and a quartz cell (1 cm path length) in a UV-Vis spectrophotometer. *Calibration curve*: In three replicates, 50.0 mg of pyrogallol was dissolved in water, immediately before use, and diluted to 25 mL. Aliquots of 1.0, 1.75, 2.5, 3.25 or 4.0 mL were diluted to 25 mL in water, and aliquots of 5.0 mL from these solutions were further diluted to 25 mL (stock solution). Each stock solution was prepared according to the procedure described above for the CE. The final concentrations were 1.28, 2.24, 3.20, 4.16 or 5.12 µg/mL of pyrogallol*.* The absorbance was measured under the same conditions as for the CE. The specific absorptivity was determined from the linear equation. The TP expressed as pyrogallol of the CE from *L. brasiliense* was calculated by Equation (1):

TP = 1562.5 A/A^1%^ m (1)

where A = Absorbance of sample for TP; A^1%^ = Specific absorptivity of pyrogallol; m = mass of the sample examined, in grams; 1562.5 = dilution factor of the sample.

### 3.7. Linearity

To establish the linearity of the proposed method, five stock solutions were prepared, in three replicates, from 10.0, 18.0, 26.0, 34.0 or 42.0 mg of CE. Concentrations of 6.40, 11.52, 16.64, 21.76 and 26.88 µg/mL were obtained.

### 3.8. Specificity

Specificity was determined by adding 1 mL of a pyrogallol solution (1.0 mg/mL) to each stock solution described in the linearity test. The method is considered specific if the slopes of the linear equations in the tests for linearity and specificity are equal or very similar.

### 3.9. Limits of Detection and Quantification

The limits of detection (LOD) and quantification (LOQ) were calculated from the relationship between the standard deviation (SD) of the CE linearity and the slope, using the appropriate multiplier. These results were compared with the LOD and LOQ obtained by extension of the linearity curve of CE: a stock solution was prepared, in three replicates, of CE (4.56 mg/mL), and aliquots of 5.0, 3.7, 2.4, 2.1, 0.2, 0.1, 0.05 and 0.025 mL were, separately, diluted to 25 mL. A reaction mixture was prepared as described in the TP method, with 2 mL from each solution. The results, together with the results for linearity, were submitted to statistical analysis. The quantification range was determined based on the lowest and highest concentrations that maintained linearity. The LOD was determined as the lowest concentration that was significantly different from the next lowest concentration.

### 3.10. Precision

Two levels were evaluated: repeatability (intra-day) and intermediate (inter-day). The repeatability was assessed using six samples of 26.0 mg of CE, and the intermediate level was assessed using three samples and on at least two different days. A coefficient of variation over 15% and a significant difference between days were considered unacceptable.

### 3.11. Accuracy

The accuracy was evaluated using different concentration levels: lowest concentration (LC), intermediate concentration (IC) and highest concentration (HC), following the addition of 1, 2, or 3 mL of pyrogallol solution (1 mg/mL) to the CE stock solution, in three replicates. Accuracy was assessed as the percentage recovery, which was calculated from Equation (2):

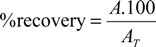
(2)
where A = absorbance of sample after addition of the standard; A_T_ = theoretical absorbance calculated for the sum of the absorbance of CE from *L. brasiliense* and the expected absorbance of pyrogallol, based on the calibration curve for each level. The method is considered accurate if the recovery percentages are between 85% and 115%.

### 3.12. Robustness

The robustness was demonstrated by analyzing the stability of the solution under the influence of natural light and a range of pH changes (7.5 and 14.06% (w/v) anhydrous sodium carbonate solutions), evaluated in three replicates.

### 3.13. Statistical Analysis

Data were analyzed by the program Statistica^®^ 8.0 (StatSoft, Inc., Tulsa, OK, USA, 1984–2007) by one-way analysis of variance (ANOVA) followed by Tukey’s test, considering *P* < 0.05 as significant. The data were expressed as mean ± standard deviation [relative standard deviation (%)]. The linear correlation tests and residual analyses were performed by simple linear regression, considering R^2^ equal to or higher than 0.98, and the residual sum of squares was evaluated.

## 4. Conclusions

After optimization of the conditions for the spectrophotometric determination of phenolics using the Folin-Ciocalteu reagent, all parameters analyzed showed adequate results. The UV-Vis spectrophotometric method described here was successfully validated as suitable for the determination of TP of CE from *L. brasiliense*. This methodology using the Folin-Ciocalteu reaction complies with the requirements for analytical use and for ensuring the reliability of the results.
